# The association between managed care enrollments and potentially preventable hospitalization among adult Medicaid recipients in Florida

**DOI:** 10.1186/1472-6963-14-247

**Published:** 2014-06-10

**Authors:** Jungwon Park, Keon-Hyung Lee

**Affiliations:** 1 Askew School of Public Administration and Policy, Florida State University, 659 Bellamy Building, Tallahassee, FL, 32306-2250, USA

**Keywords:** Preventable hospitalization, Medicaid managed care, Rurality, Competition, Spillover effect, Ambulatory care sensitive conditions

## Abstract

**Background:**

The intent of adopting managed care plans is to improve access to health care services while containing costs. To date, there have been a number of studies that examine the relationship between managed care and access to health care. However, the results from previous studies have been inconsistent. Specifically, previous studies did not demonstrate a clear benefit of Medicaid managed care. In this study we have examine whether Medicaid managed care is associated with the probabilities of preventable hospitalizations. This study also analyzes the spillover effect of Medicaid managed care into Medicaid patients in traditional FFS plans and the interaction effects of other patient- and county-level variables on preventable hospitalizations.

**Methods:**

The study included 254,321 Medicaid patients who were admitted to short-term general hospital in the 67 counties in Florida. Using 2008 hospital inpatient discharge data for working-age adult Medicaid enrollees (18-64 years) in Florida, we conduct multivariate logistic regression analyses to identify possible factors associated with preventable hospitalizations. The first model includes patient- and county-level variables. Then, we add interaction terms between Medicaid HMO and other variables such as race, rurality, market-level factors, and resource for primary care.

**Results:**

The results show that Medicaid HMO patients are more likely to be hospitalized for ambulatory care sensitive conditions (ACSCs) (OR = 1.30; CI = 1.21, 1.40). We also find that market structure (i.e., competition) is significantly associated with preventable hospitalizations. However, our study does not support that there are spillover effects of Medicaid managed care on preventable hospitalizations for other Medicaid recipients. We find that interactions between Medicaid managed care and race, rurality and market structure are significant.

**Conclusions:**

The results of our study show that the Medicaid managed care program in Florida was associated with an increase in potentially preventable hospitalizations for Medicaid enrollees. The results suggest that lower capitation rate has been associated with a greater likelihood of preventable hospitalizations for Medicaid managed care patients. Our findings also indicate that increased competition in the Medicaid managed care market has no clear benefit in Medicaid managed care patients.

## Background

Managed care is a technique that is intended to reduce the cost of providing health care and improve the quality of that care. In Florida, Medicaid enrollment grew 3.4 times as fast as population growth from 1990 through 2010 [1-3]. During the same period, Florida’s Medicaid expenditures increased at an annual rate of 10.1 percent [4]. In short, the Florida Medicaid program is the fifth largest program in terms of Medicaid spending and it ranks fourth in enrollment.

In 1984, in order to slow the rate of growth of Medicaid expenditures, Florida adopted Medicaid managed care (MMC) program options such as Health Maintenance Organizations (HMOs) and Provider Service Networks (PSNs). The HMO systems combine the financing and delivery of comprehensive health care services and supplies into one organization. PSN is “a network established or organized and operated by a health care providers or group of affiliated health care providers” [5]. Contrary to HMO, PSN is considered a mean to avoid “middleman” costs because Medicaid pays healthcare providers directly while PSN uses various managed care techniques to control utilization and cost of health care [6]. The Agency for Health Care Administration (AHCA) has primary responsibility for Florida’s Medicaid program and has contracted with HMOs on a prepaid fixed monthly rate per member since 1984. In order to expand Medicaid managed care choices, Florida implemented the Medicaid Provider Access System (MediPass) in 1991. MediPass is a statewide primary care case management program (PCCM) and it is a non-risk form of managed care. With the MediPass, primary care physicians (PCPs) provide care coordination services and disease management services to MediPass enrollees in return for a small monthly patient management fee (i.e., $2) per enrollee, plus Medicaid reimbursement for services that are rendered. On March 1, 2000, a contract between South Florida Community Care Network and AHCA officially created a Medicaid PSN in Florida [6]. As of February 2012, there are 18 Medicaid HMOs plans in Florida and 7 Medicaid PSNs as of October 2011. In 2008, Medicaid HMOs, MediPass, and Medicaid PSNs accounted for approximately 46%, 54%, and 1% of total Medicaid managed care enrollments, respectively. Currently, Florida Medicaid recipients have at least 15 different managed care program options in Medicaid and more than two-thirds (i.e., about 1.9 million) of all Florida Medicaid recipients were enrolled in one of the managed care programs [7,8].

Florida statues mandate most Medicaid recipients to enroll in a managed care plan or MediPass. However, individuals in an institution, individuals enrolled in the Medicaid medically needy program or patients receiving hospice care are exempted from managed care programs by state and/or federal law. Florida statues also allow people in exemption categories to voluntarily enroll in managed care programs, such as certain pregnant women and those dually eligible for Medicaid and Medicare. In 2011, Part IV of Chapter 409, Florida Statues, was created by the Florida Legislature to establish the Statewide Medicaid Managed Care program in which more Medicaid beneficiaries are required to enroll in a managed care program on a mandatory basis.

As noted above, the intent of adopting managed care plans is to improve access to health care services while containing costs. In an HMO, a member must select a primary care physician. Contracted primary care physicians (PCPs) are responsible for the overall care of the HMO members and act as gatekeepers. An HMO member pays a fixed monthly fee regardless of how much care they receive. As a result, HMO members have a low cost barrier to getting health care, especially to primary care, and they seek medical treatment early. However, since Medicaid recipients do not pay premiums and coinsurance, it is not plausible to say Medicaid managed care members have a lower cost barrier to getting primary care than Medicaid FFS enrollees. Thus, we were more concerned with the insurer’s incentive than the patient’s incentive in explaining the relationship between Medicaid managed care and preventable hospitalizations. Since a health maintenance organization has a strong incentive to keep cost and utilization rates low, managed care plans provide cost-containment incentives (i.e., bonuses) to health care providers. Health care providers under managed care plans emphasize preventive care since they have more financial rewards in prevention of illness than treatment of illness. In addition, using various management strategies (i.e., gatekeeper and utilization reviews), managed care plans focus on primary care services to avoid costly specialty visits and hospitalizations. Thus, it is expected that enrollees in Medicaid managed care plans have lower preventable hospitalization rates than enrollees in Medicaid FFS.

This study also assesses how rurality is related to obtaining primary care services because Medicaid recipients may face barriers to receiving primary care depending on their residence. For example, residents in rural communities are less likely to receive primary health care of reasonable quality regardless of their insurance type because of limited accessibility to primary care [9]. This geographic barrier is associated with lack of availability and quality of local primary care in rural areas. In short, an insufficient number of primary care physicians, lack of hospitals and clinics, lack of information, and long travel distances to access care characterize healthcare in rural areas.

The availability of consumer choice and competition in Medicaid managed care is different across markets. While some counties use PCCM only, other counties use both PCCM and HMO. The number of Medicaid managed care plans that counties provide is not uniform, ranging 1 to 11 across the 67 counties in Florida [10]. Also, there are different levels of competition in the Medicaid managed care markets across counties. Some Medicaid HMOs face high levels of competition. In other counties, Medicaid HMOs are in a more subdued competitive environment. These different market structures may yield different effects on access to primary care for Medicaid managed care enrollees. In addition to the direct relationship between Medicaid managed care organization market structure and preventable hospitalizations, we added the interaction between MMC enrollment and MMC market structure to assess differences in the relationship between managed care enrollment and preventable hospitalizations in the Medicaid managed care market structure. The competitive behavior of Medicaid managed care organizations is likely to be affected by different market structures. We expect that increased competition in the managed care market benefits Medicaid managed care enrollees by enhancing the quality of care. Since there is a lack of a price mechanism between Medicaid managed care organizations and their enrollees, a different quality of product—such as more comprehensive coverage and better access to providers—can affect the health care plan selection of Medicaid enrollees [11].

The purpose of this study is to investigate how managed care, county-level rurality, and market structure (i.e., number of competitors and degree of competition) of Medicaid managed care are related to potentially preventable hospitalizations. We also study the spillover of Medicaid managed care on access to primary care because growth in the managed care delivery system could affect those who are not covered by managed care plans [12,13]. The growth of managed care may affect physician practice pattern throughout the area [14,15]. To reduce preventable hospitalizations, for example, non-HMO providers could follow the HMO model [13]. In addition, the prevalence of managed care could influence the kind of services available and the “variation in the mix of services” available in their areas [12]. Finally, this article presents empirical evidence regarding the relationship between other external barriers–health care resources (i.e., the presence of federally qualified health centers and rural health clinics and physician supply, etc.) – and the likelihood of hospitalizations for ambulatory care sensitive conditions (ACSCs).

Using the 2008 hospital inpatient discharge data in Florida and 2011-2012 Area Resource File, we test multiple logistic regression models for the likelihood that a Medicaid patient will be admitted for ACSCs. Better understanding of the relationship between managed care, geographic barriers, market structure and potentially preventable hospitalizations can usefully inform policy. To date, there have been a number of studies that examine this relationship [9,16-19]. However, the results from previous studies have been inconsistent. Since 2000, five studies have examined whether Medicaid managed care affects preventable hospitalization patterns [16,20-23], but none of them analyzed the Medicaid population in Florida. Previous studies did not demonstrate a clear benefit of Medicaid managed care [16,20]. For example, Basu et al. [16] found that Medicaid managed care was not associated with a decrease in the number of preventable hospitalizations. Also, relatively little is known about how the level of rurality affects preventable hospital admissions among Medicaid adults. Only two out of five studies controlled for rurality in their analytic models [16,22]. Regarding HMO market-level factors, there has been limited research regarding the effect of levels of HMO competition on preventable hospitalizations [13].

Overall, this study contributes to the current understanding of Medicaid managed care by examining whether Medicaid managed care is related to preventable hospitalizations and how Medicaid managed care interacts with county-level characteristics and market-level factors in estimating avoidable hospitalizations. Specifically, the following research questions were addressed in the study:

1. Is Medicaid managed care enrollment related to preventable hospitalizations?

2. What is the indirect association of Medicaid managed care penetration with preventable hospitalizations of those who are not covered by a managed care plan?

3. How does the association of managed care with preventable hospitalizations vary by race, county-level characteristics, or market-level factors?

## Methods

### Data and samples

The two main databases used in this study are the AHCA hospital inpatient data file for 2008, and the Health Resources and Services Administration (HRSA) 2011-2012 Area Resource File (ARF). The AHCA hospital inpatient data file includes both clinical and non-clinical variables for each hospital stay in Florida. The HRSA ARF contains variables about county-level contextual factors. Because hospital inpatient data is patient-level data, to create an analytic file, the patient-level information obtained from inpatient discharge data is linked to county-level variables from the Area Resource File through the patient’s county. We also use the AHCA MediPass/Medicaid HMO Recipient Enrollment report and Non-reform Medicaid HMO Enrollment reports for January-July 2008 to obtain county level Medicaid enrollment data and market level measures of the Medicaid managed health care market.

The sample for this study includes Medicaid patients who were admitted to short-term general hospitals in the 67 counties in Florida. Our study focuses on the working-age adult group (18-64 years); therefore we exclude the pediatric population (0-17 years) and the elderly population (65 years and older) from this analysis. These three age groups have different “health profiles and needs, insurance availability, and health care utilization” rates [9]. Thus, these three groups may face different access barriers to primary health care. There are three basic eligibility pathways for adult Medicaid beneficiaries in Florida: Supplemental Security Income (SSI) recipients; children and families, including pregnant women; and aged, blind and disabled persons, including those needing institutional care. Among non-disabled adults, parents of dependent children in low-income families (up to 19% of the Federal Poverty Level) are also eligible for Medicaid. However, Medicaid does not cover non-disabled childless adults in Florida. Most recent Medicaid enrollment reports show that adult beneficiaries account for 38% of the Temporary Assistance for Needy Families (TANF)-related group and 45% of the SSI group. Although information about Medicaid managed care enrollment by program-group by age is not available, total Medicaid managed care enrollment by program-group shows that 76% of the TANF-related group and 81% of the SSI group enrolled in a Medicaid managed care program in Florida as of January 2008.

Since no variables are missing more than 5% of the total number of cases, the missing data is not problematic for the analysis [24]. Thus, we deleted the missing observations. After deleting the missing values, the number of observations in the sample is 254,321.

### Ethical considerations

This study uses secondary analysis of existing data that are publicly available. The hospital inpatient data from AHCA is anonymized, thus the databases did not make any of the personally identifiable information available to the researchers. As such, the study has been certified as exempt from ethical review. The determination of an exempt status was made by the Florida State University Human Subjects Committee (The Assurance Number: IRB00000446).

### Outcome measure

The outcome of interest is the hospitalizations for ambulatory care sensitive conditions. Ambulatory care sensitive conditions^a^ are “diagnoses for which timely and effective outpatient care can help to reduce the risk of hospitalization by either preventing the onset of an illness or condition, controlling an acute episodic illness or condition, or managing a chronic disease or condition” [25]. Several researchers use hospitalizations for ACSC as a measure of access to primary care [26,27]. In this study, we use ambulatory care sensitive conditions as defined by the Agency for Healthcare Research and Quality (AHRQ) [28,29]. The AHRQ provides Prevention Quality Indicators (PQIs) to identify quality of care for ambulatory care sensitive conditions. There are 14 PQIs. We use 12 of these PQIs to identify ACSCs in the adult population: diabetes short-term complications, diabetes long-term complications, chronic obstructive pulmonary disease, asthma, hypertension, heart failure, dehydration, bacterial pneumonia, urinary tract infections, angina without procedure, uncontrolled diabetes, and lower-extremity amputation. An additional file presents AHRQ’s ACSCs with codes from the International Classification of Diseases, Ninth Revision (ICD-9-CM) system [*see* Additional file 1]. Following the AHRQ instructions, we also applied several exclusion criteria based on age, sex, selected procedures, and admission types. For more information about definitions, technical specifications, and inclusion and exclusion criteria, please go to the AHRQ Web site at http://www.qualityindicators.ahrq.gov/Modules/PQI_TechSpec.aspx. If the patient’s principal diagnosis code is included in the list of PQIs, the ACSC variable are coded as 1 and all other diagnosis codes are 0.

### Explanatory variables

The insurance status of the patient is a dichotomous variable (1 = Medicaid managed care, 0 = Medicaid FFS). In this study, all three-program types (i.e., Medicaid HMOs, MediPass, and Medicaid PSNs) are considered as Medicaid managed care. We use the 2003 Urban Influence Code (UIC) from the Economic Research Services to measure the degree of rurality of a county. The UIC classifies counties based on population and commuting data, and then every county is assigned to one of twelve groups. The UIC information related to noncore counties was used to indicate the rurality of a county. Noncore counties do not have urban clusters of 10,000 or more residents. The regression model included a dummy variable for rurality. In this study, 18 of Florida’s 67 counties were designated as non-core rural counties. To determine whether the Medicaid managed health care market structure affects preventable hospitalizations we use three market-level measures from the AHCA Medicaid enrollment reports: (1) Medicaid managed care (MMC) penetration rates; (2) number of Medicaid managed care organizations (including MediPass); and (3) the Herfindahl-Hirschman Index (HHI) of Medicaid managed care markets. The rate of MMC penetration represents the level of managed care activity and is calculated by dividing Medicaid managed care enrollment data, including HMO, PSN, MediPass, by the total Medicaid enrollment in the county. Two other measures reflect market competition. First, market competition may be more intense as the number of Medicaid HMO plans increase. During the study period, the number of Medicaid managed care plans ranges 1 to 11 across the counties in Florida. While the number of Medicaid HMOs is a simple measure of competition, a HHI index takes into account the different market share of each firm competing in the market. The HHI is calculated by summing the square of each firm’s market share. The value of the HHI index ranges from 0 (pure competition) to 1 (pure monopoly), and a higher HHI value indicates a highly concentrated market. In 2008, the average Medicaid HMO HHI index of Florida was 0.673.

This study included several variables related to resources for primary health care at the county-level: (1) per capita primary care physicians and non-physician clinicians; (2) per capita short term general hospital beds; and (3) per capita federally qualified health center (FQHC) or a rural health center (RHC). These supply related variable are dichotomous variables which are coded 1 if a county has a greater than average value in each variable, otherwise 0. In general, primary care physicians consist of family medicine, pediatrics, and internal medicine. However, we exclude pediatricians because this study examines only the adult population. In addition, we include the number of non-physician clinicians (NPCs), such as physician assistants and nurse practitioners, in the regression model because NPCs engage in delivering primary health care. Patient characteristics were controlled for in the regression model using gender, race, age, and severity of illness. To control for severity of illness, the total number of comorbidities from the Present of Admission indicator was summed for each patient. Also, at the county-level, we controlled for median household income, poverty rate, unemployment rate, and total number of Medicaid admissions. In addition to the above variables, we also included two-way interaction variables between Medicaid managed care enrollment and the variables at patient- and county (market)-level to estimate the differential association between Medicaid managed enrollment and preventable hospitalizations with these moderators. At the patient level, previous studies showed that managed care enrollment has a different effect on ACSCs depending on race and ethnicity [22]. So, we included race as a moderator. In addition, rurality and county resources variables interacted with Medicaid managed care enrollment to test whether the Medicaid managed care system was more sensitive to rural residents and the availability of local health care resources (i.e., the number of PCPs and community health centers) in affecting the likelihood of preventable hospitalizations. Finally, we added interaction terms between MMC enrollment and MMC market structure variables.

### Statistical analysis

When estimating the link between managed care enrollment and preventable hospitalization rates, the Florida Medicaid mandatory enrollment policy which is based on eligibility category might cause policy endogeneity unless mandatory groups and exempted groups have similar characteristics in terms of underlying health and poverty. These unobserved factors affect the incidence of preventable hospitalizations. In addition, Florida statues allow people in exemption categories to voluntarily enroll in managed care programs, thus the relationship between Medicaid managed care enrollments and preventable hospitalizations can be hindered by the endogeneity of the enrollment decisions: healthier Medicaid beneficiaries are more likely to enroll in managed care plans and less likely to be hospitalized [30-32]. Due to limitations in the data source, we cannot fully examine the endogeneity of enrollment of Medicaid managed care, but we have sought to minimize the potential for bias resulting from omitted variables by including several individual- (i.e., age, race, and comorbidities) and county-level variables (i.e., income, poverty, unemployment rate, and rurality) To capture the difference in underlying health status between Medicaid HMO patients and Medicaid FFS patients who were admitted to hospitals, we measured patient severity (i.e., number of comorbidity) as a control variable [17].

For the analytic technique, we use multivariate logistic regression models to analyze the binary dependent variable. The following covariates were included in the models: (1) patient level: insurance status, sex, race, age, and comorbidity; and (2) county level: rurality, Medicaid managed care penetration rates, number of Medicaid HMOs, HHI of Medicaid managed care markets, health resource availability (i.e., primary care providers, hospital beds, and community health centers), income, poverty rates, unemployment rates, and the number of Medicaid hospital admissions. For analyses, we disaggregated county-level variables to the patient-level. Odds ratios and 95% confidence intervals were estimated. The data were analyzed using STATA software, version 12 (Stata- Corp. 2011. Stata Statistical Software: Release 12. College Station, TX: StataCorp LP).

This study compared two models with and without interaction terms. The first model includes patient- and county-level variables. Then, we added interaction terms between Medicaid HMOs and other variables such as race, rurality, market-level factors, and resources for primary care. In both models, standard errors were adjusted for clustering on county using Huber-White estimator of variance. Since the logit coefficient in the non-linear model cannot be used for interpreting interaction terms, we computed cross-partial derivatives to interpret the interaction terms [33,34]. To compute the cross-partial derivatives, we used ‘-margins’ command in Stata [33]. A significance level of p < .10 was considered significant for all tests.

## Results

### Characteristics of study samples

Table 1 provides the descriptive statistics for the variables used in the analysis, broken down by Medicaid managed care, Medicaid FFS, and all-working age samples. The percentage of hospitalizations for ACSC is 17.8 among Medicaid patients, and a significant proportion of Medicaid patients’ plans remain in traditional Medicaid FFS (76.7 percent). Regarding race, White accounts for 43.7 percent of inpatient discharges, and Black account for 30.5 percent of all inpatient discharges. The mean age of Medicaid patients is about 36. The average Medicaid patient has 4 comorbid conditions at admission. For county characteristics, 18 counties were noncore rural counties (26.9 percent). Almost every county has more than one community health center such as a FQHC or an RHC (97 percent). The mean numbers of primary care physicians and non-physician clinicians in the counties are 5.82 and 9.25 respectively. On average, a county has 26 short-term general hospital beds (per 10,000 adult population). The mean number of admissions per 10,000 Medicaid enrollees per year is 253. When including MediPass run by the State of Florida, Florida had a fairly large Medicaid managed care penetration in 2008. The average MMC penetration rate was about 60 percent in 2008. On average, Medicaid beneficiaries residing in Florida counties had three Medicaid managed care plans from which to choose. However, there were 32 counties which offered only one Medicaid managed care plan (i.e., MediPass). When taking into account the market share of Medicaid HMOs, the Medicaid managed care market in Florida is highly concentrated. The average HHI is 0.673. The HHI of 55 counties is above 0.25, which indicates high concentration [35]. Of the 67 counties in Florida, 32 were controlled by a single managed care plan in 2008.

**Table 1:** Characteristics of the study sample

**Characteristics**	**Medicaid**			**All working-age sample**^**1**^
	**All**	**HMO**	**FFS**	
***Patients’ characteristics (N)***	254,321	59,200	195,121	1,114,151
**Hospitalization (%)**				
ACSC admission	17.80	25.44	15.48	22.04
Non-ACSC admission	82.20	74.56	84.52	77.96
**Age group in years (%)**				
18-24	28.01	19.18	30.69	12.06
25-40	36.85	32.94	38.04	29.96
41-64	35.14	47.89	31.27	57.98
**Sex (%)**				
Male	23.09	29.82	21.05	38.43
Female	76.91	70.18	78.95	61.57
**Race (%)**				
White	43.67	40.51	44.63	60.28
Black	30.50	39.99	27.62	20.93
Others	25.83	19.50	27.75	18.79
**Number of comorbidities (%)**				
None	19.37	12.25	21.53	12.01
One	13.90	10.30	14.99	11.88
Two or more	66.73	77.45	63.47	76.11
***County characteristics***^**2**^				
**Rurality (%)**				
Rural	2.43	0.64	2.97	2.13
Urban	97.57	99.36	97.03	97.87
**MMC Penetration rates (%)**				-
< 50%	14.89	20.74	13.11	17.16
50% - 60%	7.75	6.80	8.04	9.18
60% - 70%	72.33	65.48	74.41	68.58
> 70%	5.03	6.99	4.44	5.07
**Number of Medicaid HMO (%)**				
1	11.33	2.97	13.86	12.07
2 – 5	26.14	26.18	26.12	27.50
6 or more	62.54	70.85	60.01	60.43
**HHI of MMC (%)**				
< 0.25	57.03	60.53	55.97	54.23
> 0.25	42.97	39.47	44.03	45.77
**Availability of Primary Care (%)**				
Low	13.66	11.51	14.31	13.44
High	86.34	88.49	85.69	86.56
**Availability of Hospital Beds (%)**				
Low	14.80	10.85	15.99	15.53
High	85.20	89.15	84.01	84.47
**Availability of CHC (%)**				
Low	96.66	98.44	96.12	97.08
High	3.34	1.56	3.88	2.92

^1^All working-age samples include all payer groups.

^2^County-level variables were disaggregated to the patient-level.

### Factors associated with preventable hospitalization

Table 2 shows the results of the logistic models with odds ratios (OR) and 95% confidence intervals (CI) for the odds ratios. For comparison, we also included the unadjusted odds ratio for each covariate in the analysis model. Specifically, the odds ratio is a measure of the likelihood of being hospitalized for at least one ambulatory care sensitive condition versus being hospitalized for other medical conditions (i.e., non ACSCs). To test for goodness of fit of the statistical model, we apply the Wald chi-square test. The Wald chi-square test provides a χ^2^ value of each model: (Model 1) 13093.1 with 17 degrees of freedom (df), and (Model 2) 19575.19 with 27 df. All χ^2^ values are significant at the *p* < .001 level. A significant χ^2^ test represents a good-fit for logistic models.

**Table 2:** Estimation results of logistic regression models

**Dependent variable: Hospitalization for ACSC**												
	**Unadjusted model**				**Adjusted models**^**1**^							
					**Model (1)**				**Model (2)**			
	**OR**^**3**^		**95% CI**^**4**^		**OR**^**3**^		**95% CI**^**4**^		**OR**^**3**^		**95% CI**^**4**^	
			**Lower**	**Upper**			**Lower**	**Upper**			**Lower**	**Upper**
**Patient characteristics**												
Medicaid managed care	1.86	***	1.82	1.90	1.30	***	1.21	1.40	3.56	***	2.36	5.37
Male	2.68	***	2.62	2.74	1.06	**	1.02	1.11	1.06	**	1.02	1.11
Black	1.24	***	1.21	1.27	1.16	***	1.07	1.26	1.25	***	1.15	1.36
White	0.98		0.96	1.00	0.80	***	0.70	0.91	0.88	**+**	0.77	1.01
Age, 25-40 years^2^	3.31	***	3.15	3.48	2.70	***	2.39	3.04	2.70	***	2.39	3.04
Age, 41-64 years^2^	22.92	***	21.87	24.02	11.48	***	10.22	12.91	11.43	***	10.17	12.85
No. of Comorbidities	1.27	***	1.27	1.27	1.16	***	1.15	1.17	1.16	***	1.15	1.17
**County characteristics**												
Rural	1.06	**+**	0.99	1.13	1.11		0.89	1.39	1.16		0.92	1.45
MMC penetration	0.998	***	0.998	0.999	1.00		1.00	1.00	1.00		1.00	1.00
No. of Medicaid HMO	1.02	***	1.01	1.02	0.99		0.97	1.01	1.00		0.98	1.03
HHI of MMC	0.85	***	0.82	0.89	0.81	**+**	0.64	1.01	0.92		0.70	1.19
Primary Care	1.00		0.97	1.03	0.85	***	0.78	0.93	0.88	**	0.81	0.96
Hospital Beds	1.06	***	1.03	1.09	1.00		0.91	1.10	1.00		0.89	1.13
CHC	1.01		0.96	1.07	1.01		0.84	1.22	1.01		0.82	1.24
Household Income	0.999	***	0.999	0.999	1.00		1.00	1.00	1.00		1.00	1.00
Poverty	1.02	***	1.01	1.02	1.02		0.99	1.05	1.02	**+**	1.00	1.05
Unemployment rate	0.95	***	0.94	0.96	0.98		0.94	1.02	0.99		0.95	1.03
Medicaid Admissions	0.999	**	0.999	0.999	1.00		1.00	1.00	1.00		1.00	1.00
**Interaction w/ MMC**												
× Black									0.78	***	0.69	0.88
× White									0.71	***	0.65	0.77
× Rural									0.53	*	0.29	0.97
× MMC penetration									1.00		0.99	1.00
× No. of Medicaid HMO									0.96	***	0.93	0.98
× HHI of MMC									0.49	***	0.34	0.71
× Primary Care									0.91		0.79	1.06
× Hospital Beds									0.98		0.81	1.19
× CHC									1.04		0.77	1.40

N = 254,321; OR = odds ratio; CI = confidence interval.

^1^Multivariate logistic regression, adjusting for the other factors shown in the table. The standard error is clustered at the county level.

^2^18-24 age group was used as the base level.

^3^+ p < .10, * p < .05, ** p < .01, *** p < .001.

^4^The level of significance of 0.05 (5%) is chosen.

Compared to the Medicaid FFS patients, preventable admissions were more likely to occur among Medicaid HMO patients. For the unadjusted analysis, the odds of a preventable hospitalization for a Medicaid managed care patient are 1.86 times higher than the odds of a preventable hospitalization for a Medicaid FFS patient. Holding all other variables constant, the odds of a preventable hospitalization for a Medicaid HMO patient is 1.3 times higher than the odds of a preventable hospitalization for a Medicaid FFS patient. We found that non-white patients were more likely to be admitted due to ambulatory care sensitive conditions (OR_black_ = 1.16 and OR_white_ = 0.80 in Model (1)). Also, male patient, older patients and patients with comorbidity were subject to frequent preventable hospitalizations (OR_male_ = 1.06, OR_age, 25-40_ = 2.70, OR_age, 41-64_ = 11.48 and OR_comorbidity_ = 1.16). With regard to county-level characteristics, patients living in rural counties had higher odds of preventable hospitalizations than patients living in urban counties in an unadjusted model (OR_rural_ = 1.11). However, there was no significant difference found between rural and urban counties after controlling other variables. We found that patients in counties with higher PCP and NPC ratios were less likely to be admitted for ACSCs. Other county resource variables such as hospital beds, and community health centers were found statistically insignificant in Model (1).

We did not find spillover effects of Medicaid managed care on other patients enrolled in Medicaid FFS. In this study, the Medicaid managed care penetration rate is not significantly associated with preventable hospitalizations. Also, we did not find any evidence supporting the presence of a significant interaction between Medicaid managed care (or Medicaid FFS) and MMC penetration rates. Other market structure measures–the HHI of MMC–were significant in Model (1), We find that Medicaid patients in a concentrated market (i.e., high HHI) were less likely to be admitted for an ACSC-related hospitalization than those in a competitive market (i.e., low HHI). Thus, increased competition in a Medicaid managed care market is positively associated with preventable hospitalizations.

### Interaction effects of patient- and county-level variables

**Figure 1:**
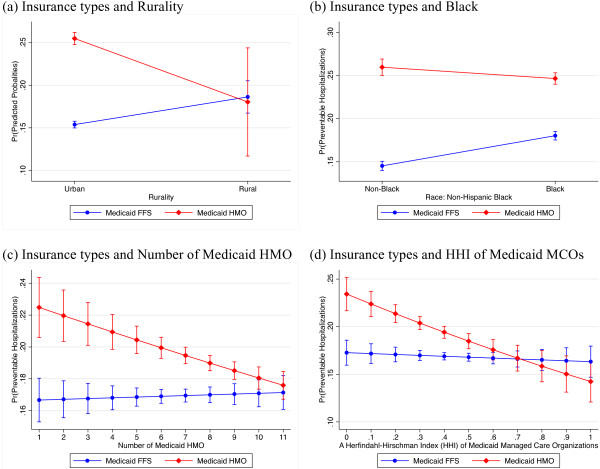
Predicted probabilities of preventable hospitalizations with 95% CIs by type of insurance (Medicaid managed care versus Medicaid FFS) and (a) rurality (b) black (c) number of Medicaid HMO (d) HHI of Medicaid MCOs.

Model (2) in Table 2 reports the interaction effects between Medicaid managed care and other patient- and county-level variables. We found five significant interaction effects – black, white, rurality, number of Medicaid managed care, and HHI of MMC. Significant interaction terms mean that the association between Medicaid managed care plan and preventable hospitalizations is different depending on the locality, race, and Medicaid managed care market structure. To interpret interaction terms in a non-linear model, a marginal effect is computed with the average marginal effect. For example, with regard to black Medicaid recipients, the odds of getting hospitalization for ACSCs for Blacks enrolled in Medicaid FFS is .35, while the odds for blacks enrolled in Medicaid managed care is .53. Thus, the marginal effect of Medicaid managed care for black Medicaid enrollees is .18. The marginal effect of Medicaid managed care for white Medicaid enrollees is .27. The marginal effect of Medicaid managed care is thus larger for White than for Black. By the same token, the marginal effect of Medicaid managed care for Medicaid enrollees in rural areas is -.05 (i.e., the odds for Medicaid FFS: .38 and the odds for Medicaid managed care: .33), while the marginal effects for Medicaid enrollees in urban areas is .25 (i.e., the odds for Medicaid FFS: .27, and the odds for Medicaid managed care: .52). Thus, Medicaid managed care is more effective in reducing preventable hospitalizations in rural areas than in urban areas. On the other hand, Medicaid managed care is positively associated with preventable hospitalizations in urban counties. Finally, the marginal effect of Medicaid managed for Medicaid enrollees in less concentrated markets (i.e., HHI < .25) is .28, and the marginal effect of Medicaid managed for Medicaid enrollees in more concentrated markets (i.e., HHI > .25) is .20. Thus, the association between Medicaid managed care and hospitalizations for ACSCs is different with respect to level of market competition. The predicted probabilities are represented graphically as seen in Figure 1.

## Discussion

In this study we have examined whether Medicaid managed care is associated with the odds of preventable hospitalizations in Florida. This study also analyzes the spillover effects of Medicaid managed care into Medicaid patients in traditional FFS plans and the interaction effects of other patient- and county-level variables on preventable hospitalizations. Our study provides important insights about the association between Medicaid managed care, geographic barriers, market structures, and potentially preventable hospitalizations in Florida.

### Medicaid managed care and preventable hospitalizations

Our findings indicate that Medicaid HMO patients are more likely to be hospitalized for ACSCs than Medicaid FFS patients. After controlling for potential confounders, the odds of preventable hospitalization for Medicaid HMO patients are 1.3 times higher than the odds of preventable hospitalization for Medicaid FFS patient. In addition, the preventable hospitalization ratio between two enrollment groups (i.e., HMO or FFS) does not correspond to the admission rate per enrollee. While Medicaid HMO enrollees (0.4%) had lower admission rates per enrollees than Medicaid FFS enrollees (1.8%), Medicaid HMO patients admitted to hospitals had higher admission rates for ACSCs than Medicaid FFS patients (43% vs. 32% of admitted patients). These results suggest that Medicaid HMO enrollees face greater barriers to accessing primary and preventive health care services than their FFS counterparts. These findings are consistent with those of Porell [20] who found potential HMO access problems. According to Porell [20], Medicaid HMO enrollees may have significant access barriers since gatekeepers in HMOs strictly control utilization. Therefore, lower hospital admission rates and higher preventable hospitalization rates for Medicaid HMO enrollees may result from a gatekeeper system and strict enforcement of utilization review.

It is also plausible that low capitation rates can limit access to care for Medicaid HMO enrollees. Florida’s reimbursement rate for Medicaid doctors is already very low compared to the national average. The average Medicaid fee in Florida was 57 percent of the Medicare rates in 2012, which was 9 percent below the national average [36]. For primary care, Florida’s Medicaid paid only 49 percent of the Medicare rate in 2012. Florida’s Medicaid primary care pay rate to Medicare levels was the 5th lowest in the U.S. Furthermore, capitation rates for risk-based Medicaid managed care programs are less than the Medicaid fee-for-services rates. Medicaid’s monthly capitated rates are based on existing Medicaid FFS spending in Florida, and AHCA applies an approximate 8 percent discount from FFS payments to control Medicaid costs [37]. Lower Medicaid reimbursement rates may decrease physician participation in Medicaid, which in turn reduces access to health care. If Medicaid payments are lower for HMOs than for FFS, physicians and hospitals may have less incentive to contract with Medicaid managed care plans. Accordingly, Medicaid HMO enrollees are more likely to suffer access-to-care problems than Medicaid FFS enrollees.

### Rurality and preventable hospitalizations

Our study shows that geographic barriers are associated with access to primary health care. The unadjusted model shows that patients living in rural counties are 1.06 times more likely than patients living in urban counties to be admitted to a hospital due to ambulatory care sensitive conditions. Our results are consistent with the results of previous studies. Previous studies have found evidence that supports the positive effect of rurality on avoidable hospitalizations [38,39]. However, after controlling for the supply of health care providers, we did not find a positive relationship between rurality and avoidable hospitalizations. The results indicate that high admission rates for ACSCs in rural counties are actually because health care resources are limited. We also assess whether the relationship between managed care and preventable hospitalizations varies depending on rurality (*see* Figure 1(a)). The results demonstrate that rural patients enrolled in Medicaid HMOs are less likely to be hospitalized for ACSCs than rural patients enrolled in Medicaid FFS. These results are consistent with the results of Long et al. [40]. Long et al. found that Medicaid managed care alleviated some access problem in rural area. Thus, Medicaid HMOs are potentially beneficial to rural residents and alleviate access-to-care problems in rural areas in Florida.

### Market structure and preventable hospitalizations

The findings from our study do not support the spillover effects of Medicaid managed care on preventable hospitalizations for other Medicaid recipients. The results show that preventable hospitalization does not relate to Medicaid HMO penetration. Also, when we examined differential association between Medicaid managed care penetration and preventable hospitalization by Medicaid managed care and Medicaid FFS enrollees, Medicaid managed care penetration made no difference between two groups.

We found that competition in the Medicaid managed care market affects access to care for Medicaid recipients. The results demonstrate that market share is an important factor in estimating risk for preventable hospitalizations. Our results show that Medicaid HMO enrollees can obtain greater benefit from a concentrated market structure. This study suggests that higher market concentration is negatively associated with hospitalization for ACSCs (*see* Figure 1(d)). It is possible that the economics of scale occur if Medicaid HMO plans increase in size. Medicaid HMO plans can reduce costs such as administrative or operative costs by achieving scale economies. As a result, HMO plans with a dominant position might increase expenditures for the care of patients and improve access to care for their enrollees. Second, managed care plans with large shares wield great market power on suppliers (i.e., physicians and hospitals). With increased market power, Medicaid HMO plans force in-network providers to offer more health care services for their members. Lastly, the dominant plans can attract and retain more health care providers in a network.

While most of the previous studies on HMO competition suggest that increased competition in managed care markets benefits HMO enrollees, our findings differ. However, our results should be interpreted with caution because government regulations on Medicaid managed care organizations might impact these differences. Government ensures that Medicaid managed care plans meet minimum standards of service to improve the quality of and access to health care. In Florida, the AHCA also set up specific standards of access to care which Medicaid managed care organizations must follow. In order to contract with a Medicaid agency, for example, managed care plans need to have “a sufficient number of PCPs to ensure adequate accessibility for enrollees of all ages” [41]. In addition, the AHCA established several other contractual guidelines to guarantee access to health care for Medicaid enrollees. If a Medicaid managed care organization fails to comply with those requirements, the Medicaid managed care organization will lose its contract. Therefore, government regulation is a key factor that determines entry into and exit from the Medicaid managed care market. With these regulations, government can control the activities of Medicaid HMOs with a dominant position.

### Study limitations

Several limitations of the study should be mentioned. First, the administrative data we use lacks information at the individual level. The administrative data does not provide information on patients’ socioeconomic status and in-depth clinical information that can influence preventable hospitalizations. Second, this study does not separate the effect of different types of managed care on the dependent variable. Actually, there are three major types of Medicaid managed health care plans in Florida: (1) HMO; (2) PSN; and (3) PCCM (MediPass). These different types of managed care plans have distinctive characteristics in terms of covered services, program design, and incentives. Thus, various types of managed care plans may work differently. However, the study does not consider differences in managed care programs because information regarding the type of managed care plan is not available in the AHCA inpatient discharge data. Third, another limitation of the study is the potential for an endogeneity bias caused by selection into managed care [17]. Although we controlled variables related to health status, endogeneity bias from other omitted variables may affect the result of current study. For example, if Medicaid enrollees with poor health conditions choose to enroll in Medicaid managed care plans (i.e. adverse selection), the risk of preventable hospitalization for Medicaid HMO enrollees would be high. Especially, in Medicaid, since selection criteria is set by and enrollment process is regulated by state and/or federal law, Medicaid managed care organizations have limited discretion in selecting their members. In addition, although certain people—for example, dual eligible and individuals with development disabilities—are exempt from mandatory managed care in Florida, people in these categories can also voluntarily enroll in Medicaid managed care. Finally, this study used only a single year of data. Cross-sectional design provides a limited basis for causal inference, especially if there are omitted variables. Since cross-sectional data cannot control for unobserved heterogeneity in the sample, further studies that use longitudinal data will provide a better understanding.

## Conclusions

The results of our study showed that the Medicaid managed care program in Florida was associated with an increase in potentially preventable hospitalizations for Medicaid enrollees. After controlling for underlying health conditions, we still found significant difference between Medicaid managed care and Medicaid FFS. The results suggest that lower capitation rate has been associated with a greater likelihood of preventable hospitalizations for Medicaid managed care patients. Our findings also indicate that increased competition in Medicaid managed care markets has no clear benefit for Medicaid managed care patients. Rather, HMO patients were better off in markets with larger HMO shares. These results suggest that Medicaid HMO plans with a large market share can better concentrate on the care of patients and improve access to care by achieving scale economics. Finally, proper monitoring activities for Medicaid managed care organizations could guarantee access to quality care for Medicaid HMO enrollees.

### Endnotes

^a^Ambulatory care sensitive hospitalizations are also known as ‘potentially preventable hospitalization’, ‘avoidable hospitalization’, or ‘unnecessary hospitalization’ and these term are used interchangeably in this study.

## Abbreviations

ACSC: Ambulatory care sensitive condition; AHCA: The Agency for Health Care Administration; AHRQ: The Agency for Healthcare Research and Quality; CHC: Community health center; FFS: Fee-for-service; FQHC: Federally qualified health center; HHI: Herfindahl-Hirschman Index; HMO: Health Maintenance Organization; MMC: Medicaid managed care; NPC: Non-physician clinician; PCCM: Primary care case management; PCP: Primary care physician; PQI: Prevention quality indicator; PSN: Provider Service Network; RHC: Rural health center; SSI: Supplemental Security Income; TANF: Temporary Assistance for Needy Families.

## Competing interests

The authors declare that they have no competing interests.

## Authors’ contributions

JP drafted the manuscript and contributed to all other aspects of the study. KHL was involved in the data collection, data analysis and performed the critical revision of the manuscript. Both authors read and approved the final manuscript.

## Pre-publication history

The pre-publication history for this paper can be accessed here:

http://www.biomedcentral.com/1472-6963/14/247/prepub

## Supplementary Material

Additional file 1 **Ambulatory care sensitive conditions and ICD-9-CM codes.** A Table shows the list of Prevention Quality Indicators defined by the Agency for Healthcare Research and Quality. Click here for file
