# Supplementary material for: The association between managed care enrollments and potentially preventable hospitalization among adult Medicaid recipients in Florida
Source: BMC Health Serv Res. 2014 Jun 10;14:247. doi: 10.1186/1472-6963-14-247 (PMC4059886; doi:10.1186/1472-6963-14-247)
Supplement: Additional file 1 — Ambulatory care sensitive conditions and ICD-9-CM codes. A Table shows the list of Prevention Quality Indicators defined by the Agency for Healthcare Research and Quality. [file 1472-6963-14-247-S1.docx]

**Ambulatory care sensitive conditions and ICD-9-CM codes**

| PQI | Description | ICD-9-CM Codes | Freq. | % |
| --- | --- | --- | --- | --- |
| Chronic conditions | |  |  |  |
| 01 | Diabetes Short-Term Complications | 250.10-250.13; 250.20-250.23; 250.30-250.33 | 1,411 | 0.55 |
| 03 | Diabetes Long-Term Complications | 250.40-250.43; 250.50-250.53; 250.60-250.63; 250.70-250.73; 250.80-250.83; 250.90-250.93; | 2,002 | 0.79 |
| 05 | Chronic Obstructive Pulmonary Disease (COPD) or Asthma in Older Adults | 466.0; 490; 491.0; 491.1; 491.20; 491.21; 491.8; 491.9; 492.0; 492.8; 494; 494.0; 494.1; 496; 493.00-493.02; 493.10-493.12; 493.20-493.22; 493.81-493.82; 493.90-493.92 | 4,769 | 1.88 |
| 07 | Hypertension | 401.0; 401.9; 402.00, 402.10; 402.90; 403.00;  403.10; 403.90; 404.00; 404.10; 404.90 (excludes cases with cardiac procedure codes, Stage I-IV kidney disease) | 1,119 | 0.44 |
| 08 | Heart Failure | 399.81; 428.0; 428.1; 428.20-428.23; 428.30-428.33; 428.40-428.43; 428.9 (excludes cases with cardiac procedure codes) | 3,290 | 1.29 |
| 13 | Angina without Procedure | 411.1; 411.81; 411.89; 413.0; 413.1; 413.9  (excludes cases with cardiac procedure codes) | 256 | 0.10 |
| 14 | Uncontrolled Diabetes | 250.02; 250.03 | 679 | 0.27 |
| 15 | Asthma in Younger Adults | 493.00-493.02; 493.10-493.12; 493.20-493.22; 493.81; 493.82; 493.90-493.92 (excludes cases with cystic fibrosis and anomalies of the respiratory system) | 895 | 0.35 |
| 16 | Lower-Extremity Amputation  among patients with diabetes | 84.10-84.19 (procedure code); 250.00-250.03; 250.10-250.13; 250.20-250.23; 250.30-250.33; 250.40-250.43; 250.50-250.53; 250.60-250.63; 250.70-250.73; 250.80-250.83; 250.90-250.93  (excludes cases with traumatic amputation of the lower extremity) | 33,808 | 13.29 |
| Acute conditions | |  |  |  |
| 10 | Dehydration | 276.5; 276.50-276.52; 276.0; 008.61-008.67; 008.69; 008.8; 009.0-009.3; 558.9; 584.5-584.9; 586; 997.5 | 1,238 | 0.49 |
| 11 | Bacterial Pneumonia | 481; 482.2; 482.30-482.32; 482.39; 482.41; 482.42; 482.9; 483.0; 483.1; 483.8; 485; 486 (excludes cases with sickle cell anemia, HB-S, or immunocompromised state) | 2,602 | 1.02 |
| 12 | Urinary Tract Infection | 590.10; 590.11; 590.2; 590.3; 590.80; 590.81; 590.9; 595.0; 595.9; 599.0 (excludes cases with kidney/urinary tract disorder, or immunocompromised state) | 2,414 | 0.95 |
|  | *Total hospitalizations for ACSCs* | | 45,267 | 17.80 |
|  | *Total hospitalizations for non-ACSCs* | | 209,054 | 82.20 |
|  | *Total hospitalizations* | | 254,321 | 100 |

Source: AHRQ Prevention Quality Indicators, Version 4.4
